# Advances in Foot-and-Mouth Disease Virus Proteins Regulating Host Innate Immunity

**DOI:** 10.3389/fmicb.2020.02046

**Published:** 2020-10-09

**Authors:** Jiangling Peng, Jiamin Yi, Wenping Yang, Jingjing Ren, Yuan Wen, Haixue Zheng, Dan Li

**Affiliations:** State Key Laboratory of Veterinary Etiological Biology, OIE/National Foot and Mouth Disease Reference Laboratory, Lanzhou Veterinary Research Institute, Chinese Academy of Agricultural Sciences, Lanzhou, China

**Keywords:** innate immunity, interferon, immunosuppression, foot-and-mouth disease virus, virus-host interactions

## Abstract

Foot-and-mouth disease (FMD) is a highly contagious disease that affects cloven-hoofed animals such as pigs, cattle, and sheep. The disease is caused by the foot-and-mouth disease virus (FMDV) which has a non-enveloped virion with icosahedral symmetry that encapsulates a positive-sense, single-stranded RNA genome of ∼8.4 kb. FMDV infection causes obvious immunosuppressive effects on the host. In recent years, studies on the immunosuppressive mechanism of FMDV have become a popular topic. In addition, studies have shown that many FMDV proteins are involved in the regulation of host innate immunity and have revealed mechanisms by which FMDV proteins mediate host innate immunity. In this review, advances in studies on the mechanisms of interaction between FMDV proteins and host innate immunity are summarized to provide a comprehensive understanding of FMDV pathogenesis and the theoretical basis for FMD prevention and control.

## Introduction

**FIGURE 1:**
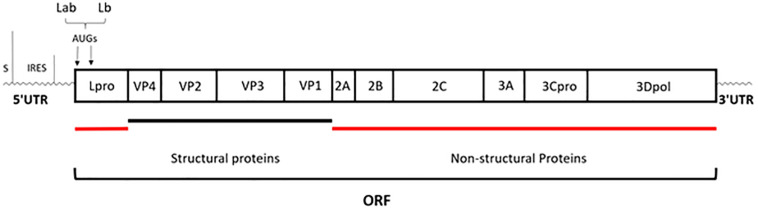
Schematic representation of genome organization. The genome of approximately 8,400 nucleotides has a single open reading frame (ORF); then is processed by the three viral proteases: L^pro^, 2A, and 3C^pro^; eventually generated four mature structural proteins (VP4, VP2, VP3, and VP1) and eight non-structural proteins (L^pro^, 2A, 2B, 2C, 3A, 3B, 3C^pro^, and 3D^pol^) (Grubman and Baxt, 2004).

Foot-and-mouth disease (FMD) is an acute, highly contagious livestock disease that affects cloven-hoofed animals such as pigs, cattle, and sheep, thereby causing severe economic loss. Typical clinical symptoms of FMD include high fever and numerous blisters on the oral mucosa, hoof, and breast, with the disease being both diverse and fast (Thomson et al., 2003). There have been several serious outbreaks of FMD in some countries, across Europe, the Middle East, Africa and Asia, and Taiwan, etc., which have hindered the development of livestock breeding and caused huge losses to the global economy (Sangare et al., 2001; Grubman and Baxt, 2004). The Foot-and-mouth disease virus (FMDV) is the pathogen that causes FMD, belonging to the *Aphthovirus* genus of *Picornaviridae* family. There are seven FMDV serotypes in the world, namely, A, O, C, South Africa 1, South Africa 2, South Africa 3, and Asia 1. Each serotype includes multiple subtypes and does not have an antigenic cross-protection reaction (Saiz et al., 2002). FMDV is a single-stranded and positive-sense RNA virus surrounded by an icosahedral capsid. Its genome, comprising of approximately 8,400 nucleotides, has a single open reading frame (ORF) that is translated into a polyprotein, which is then processed by the three viral proteases L^pro^, 2A, and 3C^pro^ into the polypeptide products P1 (VP1 to VP4), P2 (2A, 2B, and 2C), and P3 (3A, 3B, 3C^pro^, and 3D^pol^) and subsequently generated four mature structural proteins (VP4, VP2, VP3, and VP1) and eight non-structural proteins (L^pro^, 2A, 2B, 2C, 3A, 3B, 3C^pro^, and 3D^pol^) (Grubman and Baxt, 2004) (Figure 1).

**FIGURE 2:**
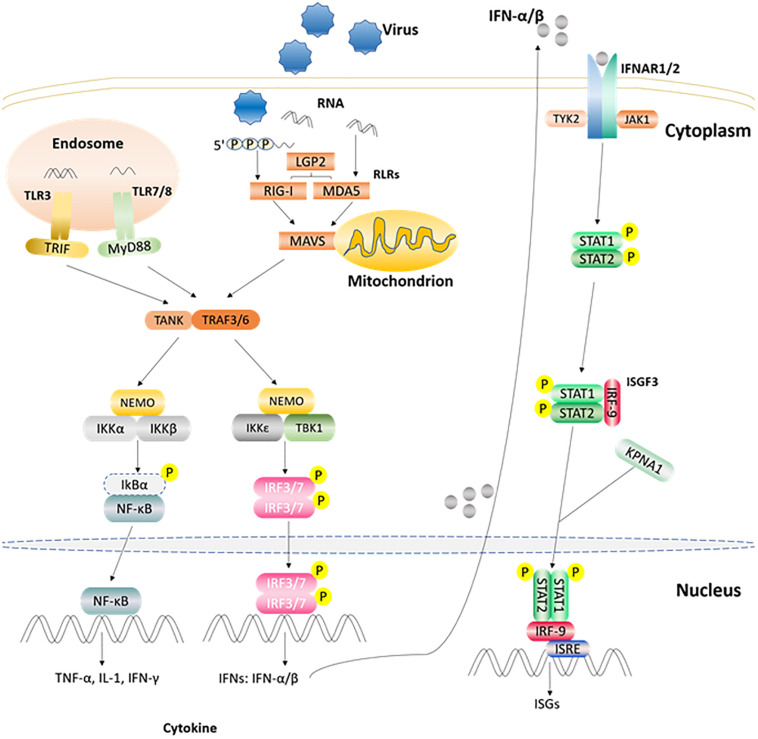
Part of the innate immune signaling pathways triggered after the viral RNA is recognized by TLRs and RLRs. After TLR3 and TLR7/8 recognize the ligand in endosome, they recruit and activate TRIF and MyD88, respectively. RLRs member recognizes corresponding ligand and activates the CARD region of MAVS. Activated TRIF, MyD88, MAVS induce TANK, and TRAF3/6. Then one signal pathway activates IKKα/β and NEMO complex, and the other activates TBKI and IKKε, NEMO complex. Subsequently, the IKKα/β and NEMO complex activates NF-κB, while TBKI and IKKε, NEMO complex phosphorylates and activates IRF3/7 to induce the expression of IFN α/β gene. The produced IFN α/β can bind to receptor IFNAR1/2, recruit STAT1/2 through the JAK1/TYK2 adaptor molecule, and recruit IRF9 through phosphorylated STAT1/2 to form the complex ISGF3 and enter the nucleus, then bind ISRE, and stimulate the expression of ISGs (Rodriguez Pulido and Saiz, 2017; Medina et al., 2018).

After the host is infected by microbial pathogens, pathogen recognition receptors (PRRs) in the host, recognize conserved molecular structures (i.e., pathogen-associated molecular patterns, PAMPs) from pathogens (Kumar et al., 2011). At present, depending on their different protein domain homologies, PRRs are classified four major families: Toll-like receptors (TLRs), RIG-I (retinoic acid-inducible-gene-I)-like receptors (RLRs), Nod-like receptors (NLRs), and C-type lectin receptors (CLRs) (Kumar et al., 2009). Once PRRs recognize PAMPs in the host cell, various anti-microbial immune responses are rapidly triggered via the induction of inflammatory cytokines, chemokines, and type I interferons (Zhong et al., 2010) (Figure 2). The innate immune system is the first line of defense against pathogens and plays a crucial role in controlling pathogen infection (Yoneyama and Fujita, 2010). Interferon (IFN) production plays a pivotal role in the host antiviral innate immune response, which can suppress viral replication especially in the early stage of the immune response. IFNs can be divided into three types according to their specific membrane-bound receptors, namely, type I, II, and III IFN (Randall and Goodbourn, 2008). Type I IFN (mainly as IFN-α and IFN-β) are more important than other interferons (such as type II, and III IFNs) in regulating the innate immune system. For example, the FMDV RNA is recognized by MDA5 and induces type I IFN production in the host to inhibit FMDV replication (Stetson and Medzhitov, 2006; Ma et al., 2018). Moreover, FMDV L^pro^ can inhibit IFN-α/β production by cleaving eIF4G and NF-κB (Hato et al., 2010). Recently, study has shown that a novel member of type I IFN, IFN-αω also has anti-FMDV activity (Li et al., 2019d). IFN-γ, the only member of the type II IFNs, can also regulate innate immunity responses (Deretic and Levine, 2018). Study has found that IFN-γ can potentially be used as a rapid method to detect the immune response of FMDV (Parida et al., 2006). Type III IFN (IFN-λ1, IFN-λ2, and IFN-λ3) are also involved in the regulation of FMDV infection. In addition to having pathogen defense functionality that is as good as that of the type I IFNs, type III IFNs can also increase adaptive immune responses in the respiratory mucosa (Ye et al., 2019). It has been demonstrated that poIFN-λ1 (novel recombinant porcine interferon lambda 1) can inhibit FMDV replication in IBRS-2 cells, and that FMDV L^pro^ has the ability to counteract this inhibition (Wang et al., 2011b). In addition, innate immune cells also play important roles in innate immune response processes, which can induce the production of IFNs in many ways to achieve antiviral effects (Golde et al., 2008). FMDV and its hosts are in a constant arms race, such that the virus has evolved multiple strategies to escape the host’s immune surveillance and defense system, which eventually destroys the balance between FMDV replication and host antiviral response (Table 1). In particular, FMDV proteins can directly or indirectly regulate the host’s innate immune response to survive and replicate in the host (Rodriguez Pulido and Saiz, 2017).

**TABLE 1:** FMDV proteins regulate and/or interact with the host’s innate immunity (Medina et al., 2018).

	**FMDV proteins**	**Viral-counter-mechanism**
Structural proteins	VP1	Interacts with host protein sorcin to inhibit I IFNs and disrupt the signal transduction of NF-κB (Li et al., 2013) Interacts with host protein DNAJA3 to reduce the antagonism on IFN-β signal pathway (Zhang et al., 2019) Acts on RPSA to conducive to the replication of FMDV (Zhu et al., 2020)
	VP3	Degrades JAK1, inhibits phosphorylation, dimerization of STAT1 (Li et al., 2016b) Inhibits expression of VISA (Li et al., 2016c) Interacts with host protein TBK1 (Li et al., 2019b)
	L^pro^	Cleaves eIF4G (Guan and Belsham, 2017) Cleaves NF-κB to reduce the production of IFN-α/β (de Los Santos et al., 2006, 2007; Hato et al., 2010) Affects the translation and transcription of IFNs (de Los Santos et al., 2006) Blocks nuclear translocation of p65/RelA (de Los Santos et al., 2007) Inhibits the transcription of IFN-β mRNA (Wang et al., 2010; Medina et al., 2018) Inhibits poly (I:C)-induced IFN-λ1 promoter activity (Wang et al., 2011b; Segundo et al., 2012) As DUB, inhibiting the ubiquitination of RIG-I, TBK1, TRAF3/6 (Wang et al., 2011a) As deISGylase, leading to the attenuation of FMDV (Medina et al., 2020) Interacts with ADNP to inhibit the expression of IFNs and ISG (Medina et al., 2017) Targets the LGP2 to reduce the level of IFNs (Rodriguez Pulido et al., 2018) Cuts G3BP1 and G3BP2 via catalytic activity (Visser et al., 2019)
Non-structural Proteins	3C^pro^	Cleaves histone H3 (Falk et al., 1990) Cleaves eIF4G and eIF4A (Belsham et al., 2000) Cuts the NEMO to inhibit the activity of IRFs and NF-κB (Wang et al., 2012a) Degrades KPNA1 and blocks the JAK-STAT pathway (Du et al., 2014) Inhibits the autophagy by degrading ATG5-ATG12 (Fan et al., 2017) Interacts Sam68 and lysate Sam68 (Lawrence et al., 2012; Rai et al., 2015)
	2B	Involves in membrane rearrangement (Moffat et al., 2005; Moffat et al., 2007) Disrupts the Ca^2+^ balance (Campanella et al., 2004; de Jong et al., 2008) Regulates LGP2 expression level to increase FMDV replication (Zhu et al., 2017) Inhibits of RLRs (MDA5 and RIG-I)-induced IFN-β production (Zhu et al., 2016; Li et al., 2018) Downregulates NOD2 to affect NF-κB and IFN-β (Liu et al., 2019) Interacts with CypA (Liu et al., 2018) Activates NLRP3 inflammasomes (Zhi et al., 2020)
	3A	Interacts with RLRs (RIG-I/MDA5/VISA) to inhibit the TLRs-mediated IFN-β signaling pathway (Li et al., 2016a) Interacts with DDX56 to reduce phosphorylation of IRF3 (Fu et al., 2019; Li et al., 2019a) Degrades G3BP1 by upregulating LRRC25 (Yang et al., 2020)

In the past two decades, thanks to the improved understanding of FMDV structure, many studies have confirmed that FMDV proteins can inhibit the production of IFNs. Therefore, this article reviews the relationship between FMDV proteins and the host’s innate immune-related proteins and also provides ideas for further studies regarding FMDV inhibiting the host’s innate immune response and regarding effective FMDV vaccines development.

## FMDV Proteins Regulate Host Innate Immunity

### The Role of FMDV Structural Proteins in the Regulation of Host Innate Immunity

Four structural proteins (VP4, VP2, VP3, and VP1) encoded by the P1 region of FMDV, mainly form the icosahedral capsid of virus particles: VP1-3 cooperate to form the capsid surface, while VP4 forms the internal structure of the virus particles. The four structural proteins differ in terms of conservation: VP1 is highly variable, VP2 and VP3 are relatively conserved, and VP4 is highly conserved among all serotypes. VP1 and VP3 play important roles in inhibiting the production of host interferons to weaken the host antiviral response. On the other hand, VP2 is involved in the induction of autophagy (Feng et al., 2018; Li et al., 2019b; Liu et al., 2020).

#### Advances in the Suppression of Innate Immunity by FMDV VP1

The FMDV structural proteins VP1, VP2, and VP3 of FMDV are folded into an eight-stranded wedge-shaped-barrel, and compose a major part of capsids; however, most of the antigen sites corresponding to the immune response are mainly found on the G-H loop of VP1 (Abubakar et al., 2018). There is a hypervariable region on the G–H loop which may lead to the high variability of VP1 (Fernandez-Sainz et al., 2019). VP1 plays crucial roles in FMDV infection, such as in inducing neutralizing antibodies, mediating cellular and humoral immunity, inducing host cell apoptosis, and promoting FMDV replication. It has been found that the FMDV structural protein VP1 can interact with soluble resistance-related calcium binding protein (sorcin) to inhibit type I IFN production induced by SeV or TNF-α, and the interaction between FMDV VP1 and sorcin can also disrupt the signal transduction of NF-κB, leading to persistent FMDV infection of the host (Li et al., 2013); however, further study is still required to determine its specific mechanism. Recently, the interaction of the host cell protein DnaJ heat shock protein family (Hsp40) member A3 (DNAJA3) with VP1 was identified through a yeast two-hybrid system. Studies has shown that DNAJA3 can significantly reduce the inhibitory effect of VP1 on SeV-induced IRF3 phosphorylation, dimerization, and nuclear localization through the lysosomal pathway, thus reducing the antagonism on the IFN-β signal pathway and inhibiting FMDV replication (Zhang et al., 2019). In addition, VP1 can also act on ribosomal protein SA (RPSA) to weaken its inhibitory effect on the mitogen-activated protein kinase (MAPK) pathway, which is conducive to the FMDV replication (Zhu et al., 2020). VP2 may indirectly inhibit the host’s type I IFN response pathway by interacting with the host protein heat shock protein family B [small] member 1 (HSPB1) to enhance viral replication, but the mechanism needs to be further elucidated (Sun et al., 2018).

#### Advances in the Suppression of Innate Immunity by FMDV VP3

Though VP3 is more conservative than VP1, it still plays an important role in the process of viral assembly and in suppressing the host innate immunity. For example, study has confirmed that arginine 56 in VP3 is relevant to FMDV virulence (Borca et al., 2012). Subsequent study found that VP3 also plays a vital role in escaping the host’s innate immune response, for example, FMDV VP3 can interact with janus kinase 1 (JAK1) and degrade it via the lysosomal pathway, thereby inhibiting IFN-γ-induced phosphorylation, signal transducer activator of transcription 1 (STAT1) dimerization and nuclear accumulation of phosphorylated STAT1, which ultimately leads to the inhibition of the type II IFN signaling pathway to evade the host innate immunity (Li et al., 2016b). Moreover, VP3 depends on its C-terminal (111–220 amino acids) to interact with virus-induced signaling adapter (VISA) and inhibits the expression of VISA by reducing VISA mRNA synthesis, finally inhibiting IFN-β production (Li et al., 2016c). Besides, FMDV VP0 protein interacts with Poly (rC) binding protein 2 (PCBP2) to degrade VISA via apoptotic pathway, thereby increasing FMDV replication (Li et al., 2019c). The latest study has found that the TANK-binding kinase 1 (TBK1) protein can interact with VP3 and degrade VP3 by its kinase and E3 ubiquitin ligase activity to resist FMDV infection (Li et al., 2019b). In addition, microRNA-1307 could enhance the host immune response by promoting VP3 degradation through the proteasome pathway in PK-15 cells to inhibit FMDV replication (Qi et al., 2019). Another structural protein, VP4, interacts with nucleoside diphosphate kinase 1 (NME1) to inhibit p53-induced activation of the IFN pathway (Feng et al., 2018).

### The Role of FMDV Non-structural Proteins in the Regulation of Host Innate Immunity

Foot-and-mouth disease virus regions P2 and P3 encode partial precursors and eight mature non-structural proteins (L^pro^, 2A, 2B, 2C, 3A, 3B, 3C^pro^, and 3D^pol^). Moreover, L^pro^, 3C^pro^, 2B, 2C, and 3A also regulate host antiviral innate immune responses.

#### Advances in the Suppression of Innate Immunity by FMDV L^pro^

The RNA translation initiation site of FMDV has two AUGs separated by 84 nucleotides. As such, there are two different forms of L^pro^ after translation, namely, Lab and Lb. It is generally believed that Lb is more powerful and more effective than Lab (Grubman and Baxt, 2004; Liu et al., 2015). L^pro^ is related to viral virulence and is obtained by self-cleavage from the C-terminus of polypeptide chain (Gys-Gly site), and is the first mature viral protein after translation (Mason et al., 2003a). Study has shown that L^pro^ can specifically cleave eukaryotic initiation factor (eIF4G) to inhibit the translation of host cap-dependent mRNA, so FMDV can destroy synthesis of the host proteins, but it does not affect FMDV mRNA translation (Guan and Belsham, 2017). In addition, L^pro^ can also cleave NF-κB, which is related to IFN-α/β production, so as to facilitate viral replication (de Los Santos et al., 2006, 2007; Hato et al., 2010). L^pro^ can affect the translation and transcription of IFNs in various ways in order to interrupt antiviral activity mediated by IFN-α/β (de Los Santos et al., 2006). L^pro^ blocks nuclear translocation of the NF-κB subunit heterodimer p65/RelA to inhibit the IFN-β expression (de Los Santos et al., 2007). L^pro^ can also reduce the protein level of IRF3/7 through its catalytic activity to inhibit the transcription of IFN-β mRNA and MDA5-mediated type I IFN (Wang et al., 2010; Medina et al., 2018). L^pro^ also inhibits poly (I:C)-induced type III IFN (IFN-λ1) promoter activity through its enzyme activity and the conserved protein domain SAP region, thereby promoting the nuclear localization of L^pro^, which is related to FMDV virulence and pathogenicity (Wang et al., 2011b; Segundo et al., 2012). As a novel type of viral DUB (deUbiquitinase), L^pro^ negatively regulates the type I IFN pathway by inhibiting the ubiquitination of innate immunity signaling molecules such as as retinoic acid-inducible gene I (RIG-I), tank-binding kinase 1 (TBK1), and TNF receptor-associated factor 3/6 (TRAF3/6) (Wang et al., 2011a). On the contrary, although L^pro^ also acts as a deISGylase, it is not important for inhibiting type I IFN, and this deISGylation activity is impaired after mutate L^pro^ W105 (the only conserved aromatic residue in all FMDV serotypes and can interact with ISG15), then leading to viral attenuation both in tissue culture and *in vivo* in mice (Medina et al., 2020). Recent study has shown that L^pro^ can inhibit the expression of IFNs and interferon-stimulated genes (ISGs) by interacting with activity dependent neuroprotective protein, a transcription factor that targets the IFN-α promoter region during early FMDV infection to increase viral replication (Medina et al., 2017); however, its mechanism still requires further study. L^pro^ can also target the innate immune molecule laboratory of genetics and physiology 2 (LGP2) to reduce the level of IFNs in infected cells, which then promotes the replication of FMDV (Rodriguez Pulido et al., 2018). In addition, L^pro^ cuts the SG scaffold proteins Ras GTPase SH3 domain binding protein 1 (G3BP1) and G3BP2 through its catalytic activity to inhibit SG formation, which may be relevant to the inhibition of the type I IFN response pathway (Visser et al., 2019). However, there are some issues about the mechanisms that are still unclear, such as the location of the cleavage site of G3BP1 and G3BP2 and the relationship between G3BP1/2 and L^pro^ inhibiting type I IFN signaling. In general, L^pro^ mainly regulates innate immunity via the aforementioned ways, so as to survive in the host and cause disease.

#### Advances in the Suppression of Innate Immunity by FMDV 3C^pro^

3C^pro^ is a proteolytic enzyme that belongs to the chymotrypsin-like cysteine protease family and plays an important role in processing viral multiprotein precursors and in viral replication (Curry et al., 2007). 3C^pro^ is highly conservative and cleaves the viral polyprotein ten times (Mason et al., 2003b). Once the mutation occurs at 3C^pro^, the original cleavage function may be inhibited, therefore, 3C^pro^ is thought to be a potential target for anti-FMDV drugs (Birtley et al., 2005; Curry et al., 2007). Furthermore, 3C^pro^ can also cleave many host proteins in infected cells to affect antiviral innate immunity. Early study has shown that 3C^pro^ is the sole viral protein that can cleave the 20 amino acids at the N-terminal of the histone H3. This cleavage may has an effect on the transcription level of cells infected with FMDV, as H3 is related to the regulatory domain of the transcriptional activity of eukaryotes, eventually almost completely destroying host cell functions (Falk et al., 1990). Based on earlier study, 3C^pro^ also has the ability to cleave eIF4G, although the cleavage site is different from the L^pro^ cleavage site, and the effect of 3C^pro^ is weaker than L^pro^. Moreover, 3C^pro^ also cleaves eIF4A in the late stage of FMDV infection, thus inhibiting the synthesis of host cell proteins (Belsham et al., 2000). Moreover, 3C^pro^ can specifically cut NF-κB essential modulator (NEMO) at the Gln 383 site due to its proteolytic activity, which inhibits the activity of IRFs and NF-κB to abrogate NEMO-mediated type I IFN signaling and increase FMDV replication (Wang et al., 2012a). The 3C^pro^ proteolytic enzyme activity is essential to induce karyopherin α1 (KPNA1, a nuclear transport receptor of phosphorylated STAT1) degradation, and disrupt the nuclear transport of STAT1/STAT2 to block the JAK-STAT pathway, which then inhibits IFN signaling (Du et al., 2014). Autophagy contributes to the antiviral effect of type I and type II IFN, whereas the virus can evade innate immunity by manipulating the autophagy pathway (Richetta and Faure, 2013). Furthermore, 3C^pro^ can inhibit autophagy by degrading ATG5-ATG12, which could positively regulate type I IFN production, in order to enhance FMDV infection (Fan et al., 2017). Previous study has shown that 3C^pro^ cleaves the C-terminal nuclear nucleotide signal of src-associated protein in mitosis (Sam68), resulting in its redistribution in the cytoplasm, and then binds to the FMDV internal ribosome entry site (IRES), thereby affecting the life cycle of FMDV (Lawrence et al., 2012). Subsequent further study has found that 3C^pro^ can interact with Sam68 to regulate FMDV replication *in vitro* (Rai et al., 2015).

#### Advances in FMDV 2B Suppressing Innate Immunity

Studies have shown that the FMDV non-structural protein precursor 2BC can block endoplasmic reticulum-golgi apparatus transport, and 2B has been found to be closely associated with the endoplasmic reticulum and found to be involved in membrane rearrangement (Moffat et al., 2005, 2007). Further research has proven that 2B is an ion channel protein composed of 154 aa and crosses the endoplasmic reticulum membrane, whereas its C-terminus and N-terminus are exposed to the cytoplasm (Ao et al., 2015). In addition, 2B has the function of increasing the host cell membrane permeability and disrupting Ca^2+^ balance in the host cell, thereby inducing autophagy (Campanella et al., 2004; de Jong et al., 2008). Moreover, 2B amino acid residues (28–147) are related to FMDV replication (Ao et al., 2015; Gladue et al., 2018). During FMDV infection, 2B can regulate the LGP2 protein expression level to increase FMDV replication (Zhu et al., 2017). Moreover, 2B can decrease the expression levels of MDA5 and RIG-I followed by disrupting the phosphorylation of key factors TBK1 and IRF3, thereby inhibiting the IFN-β production in the RLRs antiviral signaling pathway (Zhu et al., 2016; Li et al., 2018). Recent study has demonstrated that 2B affects the NF-κB and IFN-β signaling pathway by downregulating nucleotide-binding oligomerization domain 2 (NOD2) expression to enhance FMDV replication (Liu et al., 2019). In addition, interaction of 2B with the host Cyclophilin A (CypA) reduced CypA-mediated degradation of L^pro^ to promote FMDV replication (Liu et al., 2018). Study has shown that CypA is involved in regulating the HCV-induced type I IFN pathway (Bobardt et al., 2013); however, whether CypA is involved in the FMDV-induced type I IFN pathway is not clear. Additionally, the transmembrane region of 2B promotes IL-1β production by activating NLRP3 inflammasomes to inhibit FMDV replication (Zhi et al., 2020).

#### Advances in the Suppression of Innate Immunity by FMDV 3A

Foot-and-mouth disease virus non-structural protein 3A is a conserved protein consisting of 153 amino acids and is much longer than other picornavirus 3A proteins (Medina et al., 2018). FMDV 3A plays a crucial role in viral replication, distinguishing the host range of infection, and virulence. For example, the deletion of 3A residues 87–106 reduces the replication and virulence of FMDV in cattle, but not in pigs (Pacheco et al., 2013). A study has shown that 3A (1–51 amino acids) can interact with RIG-I/MDA5/VISA and inhibit the TLR-mediated IFN-β signaling pathway by downregulating their mRNA expression level, thereby escaping the host innate immunity (Li et al., 2016a). Furthermore, the interaction between FMDV 3A and DEAD-box family protein (DDX56) inhibits type I IFN signaling pathway by reducing IRF3 phosphorylation to increase FMDV replication (Fu et al., 2019; Li et al., 2019a). In addition, recent study has shown that 3A can degrade G3BP1 by upregulating the expression of autophagy-related protein leucine rich repeat-containing 25 (LRRC25) to reduce IFN-β production, thereby facilitating the replication and growth of FMDV (Yang et al., 2020).

### The Role of Other FMDV Proteins in the Regulation of Host Innate Immunity

Except for the aforementioned viral proteins that have been widely studied, there are other FMDV proteins involved in the process of regulating the host innate immune response. The non-structural FMDV protein 2C can interact with host protein Nmi (N-myc and STAT interactor), which may be involved in the FMDV 2C-induced apoptosis (Wang et al., 2012b). Further study has indicated that 2C and Nmi induce a type I IFN response, and expression of FMDV 2C or Nmi significantly suppresses VSV replication (Zheng et al., 2014). In addition, 3D^pol^ interacts with DEAD-box RNA helicase 1 (DDX1), which has been identified to inhibit FMDV replication due to its ATPase or helicase activity and induce the production of host IFN-β protein to enhance antiviral innate immunity (Xue et al., 2019). In addition, the non-coding regions (i.e., 5′Grubman and Baxt, 2004). For example, study has shown that the S fragment deletion mutants of the FMDV 5′UTR can increase mRNA levels of IFN-β and ISGs, and weaken FMDV virulence (Kloc et al., 2017). Both 3D^pol^ and ISES can also interact with Sam68 to regulate viral RNA translation (Rai et al., 2015). The host protein G3BP1 can directly interact with the IRES of FMDV 5′afterward, G3BP1 is cleaved by L^pro^ and 3C^pro^, upon which both products, namely, Ct–G3BP1 and Nt–G3BP1, can inhibit FMDV cap– and IRES–dependent translation (Galan et al., 2017). In terms of its function in enhancing IRES activity and determining virulence, the FMDV 3′UTR can bind to S fragment and IRES, and form a 5′–3′ bridge (IRES–3′UTR or S–3′UTR) (Serrano et al., 2006; Gao et al., 2016).

## Concluding Remarks and Further Perspectives

In this review, we mainly list the studies on the interaction of FMDV proteins with host proteins to regulate innate immunity. FMDV proteins can act on host proteins through a variety of ways to directly or indirectly block the innate immune signaling pathway in order to enhance its replication ability. For example, FMDV cleaves host proteins by its self-enzyme activity, and FMDV interacts with key factors in the innate immune pathway to increase its replication (Medina et al., 2018). These mechanisms have shown that FMDV disrupts the dynamic balance between virus and host, thereby providing the direction and theoretical basis for further FMDV research. Given that FMDV has a worldwide spread and seriously affects global economy and trade, FMD is listed as a class A infectious disease by the World Organization for Animal Health (OIE). Therefore, the aforementioned studies provide a solid theoretical foundation for FMDV vaccine development and technical guidance for FMD control (Aghaei et al., 2019; Dhanesh et al., 2020).

Due to the complexity of the FMDV structure and mutation-prone nature of the FMDV genome, the detailed mechanism of FMDV-mediated host’s innate immune response needs to be further investigated. Several preventive measures are not effective, leading to cross-infection of multiple serotypes, thereby making the diagnosis and prevention of FMDV more difficult. Consequently, there are still some questions that need to be explored: (1) Do the FMDV proteins have other sites to interact with host factors that suppress immune responses? (2) Are there interactions between FMDV proteins that promote FMDV replication? (3) What is the relationship between the inflammatory response and clinical symptoms caused by FMDV infection?

In summary, FMDV proteins interact with host proteins or cytokines to regulate the host’s innate and adaptive immune responses, which subsequently weakens the immunity of the animal and promotes FMDV replication as well as persistent infection. This review provided a theoretical basis for the development of FMDV vaccines and anti-FMDV drugs, as well as novel ideas regarding the diagnosis and control of FMD.

## Author Contributions

JP and DL conceived and designed the study. JP, JY, WY, JR, YW, HZ, and DL wrote the manuscript. All authors contributed to the article and approved the submitted version.

## Conflict of Interest

The authors declare that the research was conducted in the absence of any commercial or financial relationships that could be construed as a potential conflict of interest.
